# The effects of serial casting on lower limb function for children with Cerebral Palsy: a systematic review with meta-analysis

**DOI:** 10.1186/s12887-020-02122-9

**Published:** 2020-07-02

**Authors:** Nikki Milne, Michelle Miao, Emma Beattie

**Affiliations:** 1 Physiotherapy Program, Faculty of Health Sciences and Medicine, Bond University, Gold Coast, QLD 4229 Australia; 2 Department of Physiotherapy, Bond Institute of Health and Sport, Bond University, Gold Coast, Australia

**Keywords:** Cerebral palsy, Serial casting, Children, Lower limb, Function, Meta-synthesis, Meta-analysis, Ankle, Range, Hypertonicity

## Abstract

**Background:**

Lower limb serial casting is commonly used therapeutically in paediatric clinical practice with some evidence to support its efficacy. This systematic review aimed to determine the effects of serial casting in isolation or combination with other therapies for the management of lower limb dysfunction in children with Cerebral Palsy (CP).

**Methods:**

A systematic literature search was conducted in February 2019 across eight databases (PUBMED, EMBASE, CINAHL, PEDro, OTSeeker, Cochrane, Scopus and Proquest) using key terms ‘Cerebral Palsy’ and ‘serial casting’ and associated synonyms. A meta-synthesis and meta-analysis were undertaken when sufficient results were available showing the effect of serial casting on functional outcomes including: Ankle range of motion; neurological measures of hypertonicity and spasticity, functional gait measures and; gross motor function.

**Results:**

Twenty-five articles from 3219 possible citations were included. Serial casting was found to be effective for: Improving ankle dorsiflexion (DF) passive range of motion (PROM) in the immediate to short-term, decreasing hypertonicity measured by Modified Ashworth Scale (MAS) in the short-term and, enhancing functional gait outcomes in the mid-term. Serial casting with or without botulinum toxin type-A (BTX-A) did not significantly affect gross motor capacity measured by Gross Motor Function Measure (GMFM). Serial casting with pharmacological intervention achieved significantly more DF PROM than serial casting alone (MD − 3.19 degrees; 95% CI − 5.76 to − 0.62; *P* = 0.01; I^2^ = 0%), however the clinical importance of improving ankle DF PROM by an additional three degrees remains unclear.

**Conclusions:**

Lower limb serial casting, improves several outcomes relevant to lower limb function supporting its clinical use for improving DF PROM, reducing hypertonicity and improving gait in children with CP. Further research using stronger methodological study designs, is indicated to explore long-term effects of serial casting on functional lower limb outcomes such as gross motor function in children with CP. Clinicians can use this information when developing individualised treatment plans for children who have CP during shared decision-making consultations.

## Background

Cerebral Palsy (CP) is classified as a group of postural and motor disorders caused by a non-progressive lesion to the developing brain, aquired before the age of two [1, 2]. Affecting approximately 2.11 per 1000 live births worldwide and slightly less in western countries (e.g. Sweden with 1.96 per 1000 live births) [3], CP is a prevalent condition seen in the paediatric healthcare setting [4]. The impact of CP on functional capacity varies greatly as does the level of disruption to the developing brain [5]. Due to its non-progressive nature, a focus of rehabilitation by physiotherapists is managing associated primary and secondary complications, such as spasticity and contracture, which are often not present at the time of the initial injury to the brain [6]. This focus is clinically reasoned to be an initial step towards improved mobility, so that the focus of rehabilitation can progress towards more functional activity and participation goals, which are thought to be more meaningful activities to individuals with CP for their daily living [7] .

Among the different types of CP, spastic CP is the most common form consisting of approximately 85.8% of diagnoses [8]. Spasticity of a muscle is defined as a velocity-dependent resistance of a muscle to stretch [9] impacting the range of motion around a particular joint [10]. Without appropriate management, spasticity may contribute to the onset of contractures of the muscles, ligaments and tendons [11, 12], affecting loading biomechanics and causing major functional limitations [13]. For example, contracture of the gastrocnemius-soleus complex can cause equinus positioning of the foot, which adversely affects standing balance and increases energy expenditure during walking [14, 15]. Interventions focused on improving posturing of the lower limb would likely enhance gait kinematics and improve walking distance [14]. Many studies have identified the amplified functional impact that contractures, specifically contractures of the lower limb, have on ambulation and a child’s ability to complete functional everyday tasks [16, 17]. There is a clear need to identity the most appropriate and effective intervention strategies to minimize negative effects and maximize functional outcomes.

Current clinical management of spasticity and contracture in the lower limb includes conservative approaches such as the use of physiotherapy, orthoses, casting and splinting [18]. Other commonly used invasive strategies for spasticity and contracture management include neurotoxin injections such as botulinum toxin A (BTX-A) and intrathecal baclofen, as well as surgery such as selective dorsal rhizotomy (SDR) and various corrective orthopaedic operations [11, 17] including tendon lengthening procedures and single-event multilevel surgeries (SEMLS) [19, 20]. Previous studies have supported the efficacy of interventions including BTX-A, diazepam and SDR for the management of spasticity [17, 21–23]. However, some previous research findings suggest that surgery for children with spasticity, if performed at a young age, can yield undesirable and sometimes unpredictable outcomes such as recurrent equinus, infection and over-lengthening of muscle tendon units [22–24]. As a result, non-surgical interventions have been suggested clinically as temporising measures until the child is more musculo-skeletally developed [23].

In the research literature, serial casting is commonly referred to as the application of two or more successive fibreglass or plaster casts to a particular joint with efforts to increase passive range of motion (PROM) around a joint by maintaining prolonged passive stretch in the submaximal or maximal range [21, 25–27] which allows more opportunity for active ROM (AROM). Submaximal refers to the point between initial resistance to passive stretch and the point at which no further stretch is possible and is determined by the clinician as well as patient tolerance [28, 29].

A recent review of systematic reviews conducted by Novak and colleagues [17] summarized the evidence of various interventions for children with CP. Evidence was found to support the use of lower limb casting to alter body structures, however, only low-quality evidence was reported to have a positive effect on improving activity limitations as per the International Classification of Functioning, Disability and Health (ICF) model [30]. In relation to the lower limbs, increased physical activity was the most common recommendation for targeting a child’s ability to complete activities (e.g. walking) [17].

Several authors from studies cited in two previous systematic reviews [17, 25] recommended serial casting be used to improve PROM at the ankle to enhance functional outcomes in children with CP. However, because serial casting is commonly used in combination with BTX-A injections [21, 31] and other therapies [19], the isolated effects of serial casting on the lower limb and in particular short-leg walking casts have not yet been clearly identified in the research literature [32], despite it being a common therapeutic intervention in the management of children with CP [21]. In 2007, Blackmore and colleagues published a systematic review reporting on limited evidence, suggesting that serial casting is more effective in the management of secondary complications of CP than no serial casting [21]. This previously published evidence regarding the effects of serial casting alone to influence lower limb function warrants further investigation with recent additions to the empirical literature. Additionally, Blackmore et al. acknowledge that many studies included in their review had insufficient sample sizes, lacked randomization and lacked blinding, making it difficult to identify the true effects of the interventions [21]. For this reason, it is important to ensure that conclusions regarding the effect of serial casting on the lower limb are based on studies with sound methodological quality. More recently two systematic reviews [32, 33] explored the empirical literature to determine the efficacy of using adjunct therapies to improve outcomes after BTX-A injections in children with CP. Whilst both systematic reviews are valuable contributions to the research literature, the aims and findings of these reviews are impacted by the contribution of BTX-A and do not set out to explore the effects of serial casting independent of pharmacological interventions such as BTX-A.

Despite the conclusions of previously published systematic reviews, it is still unclear as to whether serial casting is effective for improving lower limb function (including the body function and structure and activity level functions such as gait and gross motor proficiency) in children with CP. This uncertainty is attributed to a wide range of study designs, casting protocols, and combinations of additional therapies documented in the current literature, making it difficult to isolate and determine the efficacy of serial casting as an intervention [17, 25] independent of pharmacological therapies. Considering recent contributions to the empirical literature regarding the potentially damaging effects of BTX-A to gait in children with CP [34], the findings from this systematic review are warranted to assist clinicians, parents and clients in shared decision-making processes. As such, the purpose of this systematic review was to critically appraise and synthesize the empirical literature regarding the effects of serial casting, compared to serial casting with pharmacological intervention, on the different variables influencing function of the lower limbs, in children with CP. In synthesizing the findings from moderate to high quality studies, the authors of this review aimed to:

1. Determine the effects of serial casting as a therapeutic intervention in isolation or in combination with other therapies (which may include pharmacological intervention) on lower limb dysfunction in children with CP.
2. Determine the effects of serial casting at different points in time post cast removal.
3. Determine if the addition of pharmacological intervention enhanced the effects of serial casting on lower limb function.

By expanding on previous reviews as well as including findings of recently published studies, this review may assist clinicians and families with clinical decision-making regarding the selection of serial casting as a possible intervention modality. For this review, passive range of motion (PROM) of the ankle is considered the critical outcome of interest, however, other important outcomes impacting lower limb function will be investigated, as will adverse events relating to serial casting protocols.

## Methods

### Literature searches

The systematic review protocol was registered in PROSPERO in 2017 (Protocol Number: CRD42017077841). The systematic literature search was initially conducted across eight databases (PUBMED, EMBASE, CINAHL, PEDro, OTSeeker, Cochrane, Scopus and Proquest) on the 8th November 2017 and updated on the 25th February 2019. A search strategy was developed in consultation with an experienced health sciences librarian around the concepts of “Cerebral Palsy” and “serial casting”, including the use of MeSH terms, and the equivalent terms on other databases. For sources which only allowed simple searches, the key terms “Cerebral Palsy”, “Splint*” and “Cast*” were utilized. Table 1 includes the comprehensive search strategy.

**Table 1:** Search Strategy

Search #	Search Strategy	Source	# Articles
1	(‘cerebral palsy’ OR (neurologic* AND condition*) OR (brain AND (injury OR injuries)) OR “Cerebral Palsy”[Mesh]) AND (splint* OR cast* OR “Casts, Surgical”[Mesh])	Pubmed	835
2	(‘cerebral palsy’:ti,ab OR (neurologic* AND condition*):ti,ab OR (brain AND (injury OR injuries)):ti,ab OR ‘Cerebral Palsy’/exp) AND (splint*:ti,ab OR cast*:ti,ab OR ‘Casts, Surgical’/exp)	EMBASE	888
3	(‘cerebral palsy’ OR (neurologic* AND condition*) OR (brain AND (injury OR injuries))) AND (splint* OR cast*)	CINAHL	218
4	Cerebral palsy splint*	PEDro	86
5	Cerebral palsy cast*	PEDro	27
6	cerebral palsy OR (Brain AND injury) OR (Brain AND injuries) OR (Neurological AND condition) OR (Neurological and conditions) AND splint* OR cast*	OTSeeker	39
7	(“‘cerebral palsy’”OR (neurologic* AND condition*) OR (brain AND (injury OR injuries)) OR [mh “Cerebral Palsy”]) AND (splint* OR cast* OR [mh “Casts, Surgical”])	Cochrane (CENTRAL) Trials only	100
8	TITLE-ABS((“cerebral palsy” OR (neurologic* AND condition*) OR (brain AND (injury OR injuries))) AND (splint* OR cast*))	Scopus	737
9	(“cerebral palsy” OR (neurologic* AND condition*) OR CP OR (brain AND (injury OR injuries))) AND (splint* OR cast*)	Proquest Health & Medical Complete	289

### Eligibility criteria

A comprehensive list of inclusion and exclusion criteria is provided in Table 2. For this review, serial casting was defined as the application or intent to apply two or more successive fibreglass or plaster casts to a targeted joint with efforts to increase PROM by maintaining prolonged passive stretch in the submaximal or maximal range [21, 25–27], which may also allow for improved AROM. Studies with an intervention that met this definition in isolation, or in combination with other interventions were included. However, objective outcome measures used in clinical practice pertaining to the lower limb were required to be provided in results for both pre-intervention and post-intervention, or if post-intervention scores were not reported, a change score needed to be documented. The primary outcome measure deemed critical for this review was ankle PROM. Other important outcomes that were justified by the physiotherapist reviewers to be commonly used in clinical practice, for measuring lower limb body function or structures which impact activity or participation levels, were included (e.g. hypertonicity, spasticity). Additionally, direct measures of activity or participation requiring lower limb involvement were included (i.e. functional gait measures, gross motor skills).

**Table 2:** Eligibility criteria for screening and selection

**Inclusion Criteria**	1. Study must be an intervention or observational study design. 2. Study must be from a peer-reviewed journal. 3. More than 80% of participants in the intervention group must have CP (or the data for the participants with CP can be isolated). 4. Participants must be human. 5. Participants must be children between the age of 0–18 years. 6. Participants must have undergone an intervention including serial casting of the lower limb (ankle, knee or hip).
**Exclusion Criteria**	1. Studies not reporting pre- and post-casting data for outcome measures. 2. Studies published prior to 1970. 3. Studies not available in a full text version. 4. Studies not published in English or an English full text version was not obtainable.

### Study selection

**Fig. 1:**
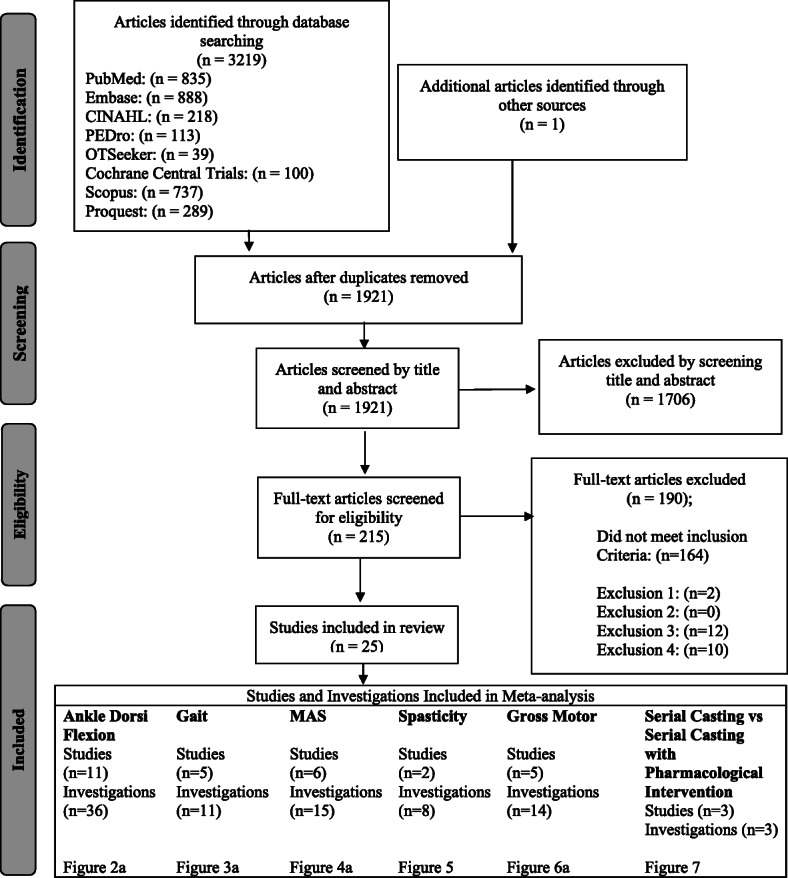
Search, screening and selection process as per PRISMA protocol https://figshare.com/s/07d850b9e6873cb3f06f

Two reviewers (EB and MM) independently ran the search and exported results to an electronic reference management software program, EndNote version X8 [35]. This software was used to identify duplicates and relevant articles, as per the PRISMA protocol [36]. As documented in Fig. 1, studies were screened by title and abstract, applying inclusion and exclusion criteria to identify records that required full text for eligibility assessment. Attempts were made to contact study authors directly via email or research platforms, regarding any data that was unclear, unpublished or unavailable in English full text. Requests were followed up on one additional occasion, after which time, if a full text version was still unavailable, studies were not included based on either exclusion criteria number three or four (see Table 2). Reviewers independently applied exclusion criteria to the remaining available full text articles, documenting reasons for exclusion. Additionally, when the same research groups had published more than one article, each reviewer independently examined the study samples and interventions to ensure independent study samples were being analysed. Final lists of eligible studies were compared, and any discrepancies were discussed amongst the two primary reviewers (EB and MM) and a third reviewer (NM) was utilised to achieve consensus when needed.

### Critical appraisal of methodological quality

The methodological quality of included studies was evaluated using the modified Downs and Black protocol [37], which addresses risk of bias. Two reviewers (EB and MM) independently determined the critical appraisal scores of each article after which, the two reviewers met to record and discuss discrepancies. When consensus on the appropriate score could not be met, a third reviewer (NM) was consulted and the consensus score was recorded. The Kennelly Rating Scale [38] was then applied to the Downs and Black Critical Appraisal Scores (CAS) (out of a possible score of 28) so that the methodological quality of each study could be determined as good (≥20 or ≥ 71%), fair (15–19 or 51–70%) or poor (≤14 or ≤ 50%) [38].

### Data extraction and synthesis

The data collected and provided in Table S1 (Supplement File 1) includes study population (age, gender, CP classification when provided), study aims, study design, intervention protocol, including intervention other than serial casting. Any additional pharmacological intervention (i.e. oral – e.g. baclofen, clonidine, tizanidine, benzodiazepines, gabapentin and dantrolene or interventional – e.g. BTX-A, phenol and intrathecal baclofen) was also extracted from studies. Outcome measures used (relevant to lower limb function), key statistical findings including the statistical analysis performed and conclusions of the publication were also reported in Table S1 (Supplement File 1). All data were extracted independently by two reviewers (EB, MM) and when discrepancies existed, a third author (NM) was engaged to independently review the data and assist with reaching agreement on important data to extract. Finally, any documented adverse events were recorded and reported in the results section of this systematic review.

A meta-synthesis was conducted to explore major trends from all included studies regarding the effects of serial casting on the lower limb in children with CP (See Table 3). Table 4 further syntheses of the effects of serial casting on outcomes relevant to lower limb function in children when the following requirements were met: i) the study was of fair or good methodological quality; ii) a minimum of five investigations had initially been published using that outcome across all included studies of fair to good methodological quality. When less than five investigations were reported using a relevant outcome measure, it was not included in the initial meta-synthesis table (see Table 3).

**Table 3:** Effects of lower limb serial casting (inclusive of additional therapeutic interventions): a meta-synthesis of findings from individual investigations reported in fair to good methodological quality studies

Outcomes relevant to lower-limb function.	Authors (reference no.) and Investigation Methods Relevant to the Effect of Serial Casting		Percentage of investigations showing a significant effect from serial casting n/N (%)	Summary Effect (+,-,0,?)
	Significant Effect (n)	Non-Significant Effect		
**Lower Limb Passive Range of Motion**				
**Ankle Dorsiflexion with Knee Flexed (KPF)**	• Cameron (45) - (SC + T + A) 1mo (+) • Cameron (45) - (SC + T + A) 4 mo (+) • Kelly (52) - (SC + P) IP (+) • Kelly 2018 (60) - (SC + P) IP (+) • Kelly 2018 (60) - (SC + P) 1 mo (+) • Kelly 2018 (60) - (SC + P) 2 mo (+) • McNee (39) - (SC) 2-3wk (+) • McNee (39) - (SC) 9-10wk (+)	• Corry (46) - (SC + T) 2wk (0) • Corry (46) - (SC + T) 3mo (+) • Kelly 2018 (60) - (SC + P) 6 mo (+)	8/11 = 72.72%	(+)
**Ankle Dorsiflexion** **with Knee Position Extended (KPE)**	• Cameron (45) - (SC + T + A) 1mo (+) • Cameron (45) - (SC + T + A) 4 mo (+) • Corry (46) - (SC + T) 2wk (+) • Kay (31) - (SC + A) 8wk (+) • Kay (31) - (SC + P + A) 8wk (+) • Kelly (52) - (SC + P) IP (+) • Kelly 2018 (60) - (SC + P) IP (+) • Kelly 2018 (60) - (SC + P) 1 mo (+) • Kelly 2018 (60) - (SC + P) 2 mo (+) • McNee (39) - (SC) 2-3wk (+)	• Corry (46) - (SC + T) 3mo (0) • McNee (39) - (SC) 9-10wk (−) • Kelly 2018 (60) - (SC + P) 6 mo (+)	10/13 = 76.92%	(+)
**Ankle Dorsiflexion with Knee Position Unspecified (KPU)**	• Boyd (15) - (SC + P + T + A) 22wk (+) • Brouwer 1998 (44) - (SC) IP (+) • Brouwer 1998 (44) - (SC) 6wk (+) • Dursun (11) - (SC + P + T) IP (+) • Dursun (11) - (SC + P + T) 9wk (+) • Flett (22) - (SC + A) 1mo (+) • Flett (22) - (SC + A) 3mo (+) • Flett (22) - (SC + A) 5mo (+) • Glanzman (16) - (SC) PE (+) • Glanzman (16) - (SC + P) PE (+) • Newman (53) - (Immediate serial casting after BTXA) (SC + P + T) 3mo (+) • Newman (53) - (Delayed serial casting after BTXA) (SC + P + T) 3mo (+) • Newman (53) - (Delayed serial casting after BTXA) (SC + P + T) 6mo (+) • Yap (58) - (SC + P + T) ~2mo (+) • Booth (28) - (SC) 3.5 wk. (+) • Booth (28) - (SC + P) 2 wk. (+)	• Boyd (15) - (SC + P + T + A) 10wk (+) • Newman (53) - (Immediate serial casting after BTXA) (SC + P + T) 6mo (+)	16/18 = 88.89%	(+)
**Functional Gait**				
**Velocity**	• Kelly (52) - (SC+ P) IP (−) • Cameron (45) - (SC + T + A) 4 mo (+)	• McNee (39) - (SC) 2-3wk (+) • McNee (39) - (SC) 9-10wk (−) • Cameron (45) - (SC + T + A) 1mo (−) • Kelly 2018 (60) - (SC + P) 1 mo (+) • Kelly 2018 (60) - (SC + P) 2 mo (+) • Kelly 2018 (60) - (SC + P) 6 mo (+)	1/8 = 12.50%	(0)
**Stride Length**	• Cameron (45) - (SC + A + T) 4 mo (+)	• Cameron (45) - (SC + T + A) 1mo (+) • McNee (39) - (SC) 2-3wk (+) • McNee (39) - (SC) 9-10wk (+) • Kelly (52) - (SC + P) IP (−)	1/4 = 25.00%	(0)
**Observational Gait Scale (OGS)**	• Dursun (11) - (SC + P + T) IP (+), • Dursun (11) - (SC + P + T) 9wk (+) • Newman (53) - (Immediate serial casting after BTXA) (SC + P + T) 3mo (+) • Newman (53) - (Delayed serial casting after BTXA) (SC + P + T) 3mo (+) • Newman (53) - (Delayed serial casting after BTXA) (SC + P + T) 6mo (+)	• Newman (53) - (Immediate serial casting after BTXA) (SC + P + T) 6mo (+)	5/6 = 83.33%	(+)
**Physicians rating scale (PRS) / modified PRS**	• Flett (22) - (SC + A) 1mo (+) (modified PRS) • Flett (22) - (SC + A) 3mo (+) (modified PRS) • Flett (22) - (SC + A) 5mo (+) (modified PRS) • Yap (58) - (SC + P + T) ~2mo (+) (modified PRS)	• Corry (46) - (SC + T) 2wks (+) • Corry (46) - (SC + T) 3mo (+)	4/6 = 66.67%	(+)
**Neurological Measures**				
**Modified Ashworth Scale (MAS) Ankle Plantar Flexors / Hamstrings**	• Dai (48) - (SC + P + T) IP (+) • Dai (48) - (SC + P + T) 6wk (+) • Dursun (11) - (SC + P + T) IP (+) • Dursun (11) - (SC + P + T) 9wk (+) • Flett (22) - (SC + A) 1mo (+) • Flett (22) - (SC + A) 3mo (+) • Flett (22) - (SC + A) 5mo (+) • Kay (31) - (SC + A + T) 8wk (+) • Kay (31) - (SC + A + T + P) 8wk (+) • Kelly (52) - (SC + P) IP (+) (Ankle Plantar Flexors) • Kelly (52) - (SC + P) IP (+) (Hamstrings) • Kelly 2018 (60) - (SC + P) IP (+) • Kelly 2018 (60) - (SC + P) 1 mo (+) • Kelly 2018 (60) - (SC + P) 2 mo (+) • Yap (58) - (SC + P + T) ~2mo (+)	• Kelly 2018 (60) - (SC + P) 6 mo (+) • Corry 1998 (46) - (SC + T) 2wks (+) • Corry 1998 (46) - (SC + T) 3mo (0)	15/18 = 83.33%	(+)
**Modified Tardieu Scale (MTS)** **(Ankle Plantar Flexors and Hamstrings)**	• Kelly (52) - (SC + P) IP (+) in Hamstrings (KE) • Newman (53) - (Immediate serial casting after BTXA) (SC + P + T) 3mo (+) in Ankle Plantar Flexors • Newman (53) - (Delayed serial casting after BTXA) (SC + P + T) 3mo (+) in Ankle Plantar Flexors • Newman (53) - (Delayed serial casting after BTXA) (SC + P + T) 6mo (+) in Ankle Plantar Flexors • Kelly 2018 (60) - (SC + P) IP (+) DF Knee Ext. • Kelly 2018 (60) - (SC + P) 1 mo (+) DF Knee Ext. • Kelly 2018 (60) - (SC + P) 2 mo (+) DF Knee Ext.	• Kelly (52) - (SC + P) IP (+) Ankle Plantar Flexors with KE & KF • Newman (53) - (Immediate serial casting after BTXA) (SC + P + T) 6mo (+) in Ankle Plantar Flexors • Kelly 2018 (60) - (SC + P) 6 mo (+) DF Knee Ext.	7/10 = 70.00%	(+)
**Gross Motor Function**				
**GMFM**	• Dai (48) - (SC + P+ T) IP (+) • Dai (48) - (SC + P+ T) 6wk (+) • Kelly (52) - (SC + P) IP (+) • Yap (58) - (SC + P + T) ~2mo (+) • Flett (22) - Standing Dimension of GMFM only (SC + A) 1mo (+) • Flett (22) - Standing Dimension of GMFM only (SC + A) 3mo (+) • Flett (22) - Standing Dimension of GMFM only (SC + A) 5mo (+) • Flett (22) – Dynamic Dimension of GMFM only (SC + A) 1mo (+) • Flett (22) – Dynamic Dimension of GMFM only (SC + A) 3mo (+) • Flett (22) - Dynamic Dimension of GMFM only (SC + A) 5mo (+) • Kay (31) - Dimensions C,D,E of GMFM only (SC + A + T) 8wk (−) • Kelly 2018 (60) - (SC + P) 1 mo (+) • Kelly 2018 (60) - (SC + P) 2 mo (+) • Kelly 2018 (60) - (SC + P) 6 mo (+)	• Kay (31) - Dimensions C,D,E of GMFM only (SC + A + T + P) 8wk (+)	14/15 = 93.33%	(+)

Intervention Coding: *SC* serial casting in isolation, + *T* plus allied therapies, + A = plus AFO / night splints / other orthotics, + *P* = plus pharmacology (which in all instances was BTX-A)

Timeframe Coding: *IP* immediately post-cast removal (within 0–1 weeks); 3mo = 3 months post-cast removal; 6mo = 6 months post-cast removal; 12 mo = 12 months post-cast removal, (time) = 1st post-test if not immediately post, *PE* peak effect time not stipulated

Individual Investigation Coding: (+) = positive effect; (−) = negative effect; (0) = nil effect

Summary Coding: n/N for Outcome (%): *n* = number of investigations with a significant positive effect, N = total number of investigations that explore the effect of serial casting on a given outcome. Summary Effect (+,-,0,?): (+) = ≥60% of investigations have a significant positive effect, (−) = ≥60% of investigations have a significant negative effect, (0) = ≥60% of investigations have a non-significant effect

**Table 4:** Effects of lower limb serial casting without pharmacological intervention: a meta-synthesis of findings from individual investigations reported in fair to good methodological quality studies

Outcomes relevant to lower-limb function.	Authors (reference no.) and Investigation Methods Relevant to the Effect of Serial Casting without pharmacological intervention		Percentage of investigations showing a significant effect from serial casting n/N (%)	Summary Effect (+,-,0,?)
	Significant Effect (n)	Non-Significant Effect		
**Lower Limb Passive Range of Motion**				
**Ankle dorsiflexion with Knee Flexed (KPF)**	• Cameron (45) - (SC + T + A) 1mo (+) • Cameron (45) - (SC + T + A) 4 mo (+) • McNee (39) - (SC) 2-3wk (+) • McNee (39) - (SC) 9-10wk (+)	• Corry (46) - (SC + T) 2wk (0) • Corry (46) - (SC + T) 3mo (+)	4/6 = 66.67%	(+)
**Ankle dorsiflexion** **with knee position extended (KPE)**	• Cameron (45) - (SC + T + A) 1mo (+) • Cameron (45) - (SC + T + A) 4 mo (+) • Corry (46) - (SC + T) 2wk (+) • Kay (31) - (SC + A) 8wk (+) • McNee (39) - (SC) 2-3wk (+)	• Corry (46) - (SC + T) 3mo (0) • McNee (39) - (SC) 9-10wk (−)	5/7 = 71.43%	(+)
**Ankle dorsiflexion with knee position unspecified (KPU)**	• Brouwer 1998 (44) - (SC) IP (+) • Brouwer 1998 (44) - (SC) 6wk (+) • Flett (22) - (SC + A) 1mo (+) • Flett (22) - (SC + A) 3mo (+) • Flett (22) - (SC + A) 5mo (+) • Glanzman (16) - (SC) PE (+) • Booth (28) - (SC) 3.5 wk. (+)		7/7 = 100%	(+)
**Functional Gait**				
**Velocity**	• Cameron (45) - (SC + T + A) 4 mo (+)	• McNee (39) - (SC) 2-3wk (+) • McNee (39) - (SC) 9-10wk (−) • Cameron (45) - (SC + T + A) 1mo (−)	1/4 = 25.00%	(?)
**Stride length**	• Cameron (45) - (SC + A + T) 4 mo (+)	• Cameron (45) - (SC + T + A) 1mo (+) • McNee (39) - (SC) 2-3wk (+) • McNee (39) - (SC) 9-10wk (+)	1/4 = 25.00%	(?)
**Physicians rating scale (PRS) / modified PRS**	• Flett (22) - (SC + A) 1mo (+) (modified PRS) • Flett (22) - (SC + A) 3mo (+) (modified PRS) • Flett (22) - (SC + A) 5mo (+) (modified PRS)	• Corry (46) (SC + T) 2wks (+) • Corry (46) (SC + T) 3mo (+)	3/5 = 60.00%	(+)
**Neurological Measures**				
**Modified Ashworth Scale (MAS) Ankle Plantar Flexors**	• Flett (22) (SC + A) 1mo (+) • Flett (22) (SC + A) 3mo (+) • Flett (22) (SC + A) 5mo (+) • Kay (31) (SC + A + T) 8wk (+)		4/4 = 100.00%	(?)
**Gross Motor Function**				
**GMFM**	• Flett (22) - Standing Dimension of GMFM only (SC + A) 1mo (+) • Flett (22) - Standing Dimension of GMFM only (SC + A) 3mo (+) • Flett (22) - Standing Dimension of GMFM only (SC + A) 5mo (+) • Flett (22) – Dynamic Dimension of GMFM only (SC + A) 1mo (+) • Flett (22) – Dynamic Dimension of GMFM only (SC + A) 3mo (+) • Flett (22) - Dynamic Dimension of GMFM only (SC + A) 5mo (+) Kay (31) - Dimensions C,D,E of GMFM only (SC + A + T) 8wk (−)		7/7 = 100%	(+)

Intervention Coding: *SC* serial casting in isolation, + T = plus allied therapies, + A = plus AFO / night splints / other orthotics

Timeframe Coding: *IP* immediately post-cast removal (within 0–1 weeks); 3mo = 3 months post-cast removal; 6mo = 6 months post-cast removal; 12 mo = 12 months post-cast removal, (time) = 1st post-test if not immediately post, *PE* peak effect time not stipulated

Individual Investigation Coding: (+) = positive effect; (−) = negative effect; (0) = nil effect

Summary Coding: n/N for Outcome (%): *n* = number of investigations with a significant positive effect, N = total number of investigations that explore the effect of serial casting on a given outcome. Summary Effect (+,-,0,?): (+) = ≥60% of investigations have a significant positive effect, (−) = ≥60% of investigations have a significant negative effect, (0) = ≥60% of investigations have a non-significant effect

Many clinicians consider an improvement in gait pattern to be a primary aim of serial casting [39], with ankle range of motion (PROM) and ankle angle at initial contact during gait noted to be critical measures affecting lower limb function that are commonly used in the clinical setting, and consequently these kinematic measures were included for investigation in this systematic review. The post-casting timeframe was calculated and recorded as the amount of time passed between the removal of the final cast and the post-casting assessment. Within a single study, comparisons between baseline and post-casting measures of a reported outcome were treated as separate investigations if the outcome data was presented at multiple post-casting timeframes. Once investigations were categorized based on outcome measures and time points for measurement, investigations were identified as having significant or non-significant findings (see Tables 3 and 4). Two stages of coding were undertaken. Individual investigations were coded based on whether the effect was positive/desirable (+), negative/undesirable (−) or no effect (0). For each outcome, the percentage of investigations reporting a significant effect (n) were divided by the total number of investigations (N) (with fair to good methodological quality) to determine the overall trends of serial casting on lower limb function. The summary effect, which was the second stage of coding, was then reported to determine the overall effect of serial casting as either positive/desirable (+), negative/undesirable (−) or no (0) effect. To determine a positive/desirable (+) summary effect, over 60% of investigations needed to demonstrate a significant positive effect. To determine a negative/undesirable (−) summary effect, over 60% of investigations needed to demonstrate a significant negative effect. If over 60% of investigations demonstrated no significant effect, it was coded as zero (0). If more than one investigation and less than five investigations were undertaken for the effect of serial casting on any outcome or it did not meet the above criteria, then it was coded as questionable (?) and reported in the narrative synthesis.

### Data analysis

To quantitatively address the three study aims, meta-analyses were conducted. These analyses were completed to assess the effect of serial casting on outcomes where a minimum of two investigations were reported in studies of fair to good methodological quality. Effect sizes were calculated as mean differences (MD) or standardised mean differences (SMD) depending on whether the data were continuous in nature or if different scales had been used. For each outcome assessed, variance was approximated using the standard deviation (SD) of the mean difference (MD) between the initial measure at baseline and the follow-up measurement. When this data was not available the SD was calculated using the Cochrane calculator [40] from the *p*-value for the differences between mean values [41]. If the p-value was not available, the highest SD available from other included studies using the same measure in this review was imputed using methods previously published [42] and this was required for ten investigations during meta-analyses. Heterogeneity was calculated and determined using Revman software [40]. Heterogeneity of the studies was assessed using the I^2^ value from the X^2^ test and was determined to be minimal when values were between 0 and 30%, moderate when reaching 31 to 50%, substantial when reaching 51 to 90% and considerable if over 90% [42]. Where heterogeneity was deemed to be significant, meta-analysis was undertaken using the random-effects model. Furthermore, the overall effects were estimated only amongst groups of investigations that used the same outcome variables and interventions met the predefined definition of serial casting. Whilst not a definitive form of assessment, graphical assessment for publication bias was undertaken for each meta-analysis using funnel plots as no unpublished data was available to ascertain with more certainty if publication bias was influencing the outcomes of each meta-analysis [43].

**Fig. 2:**
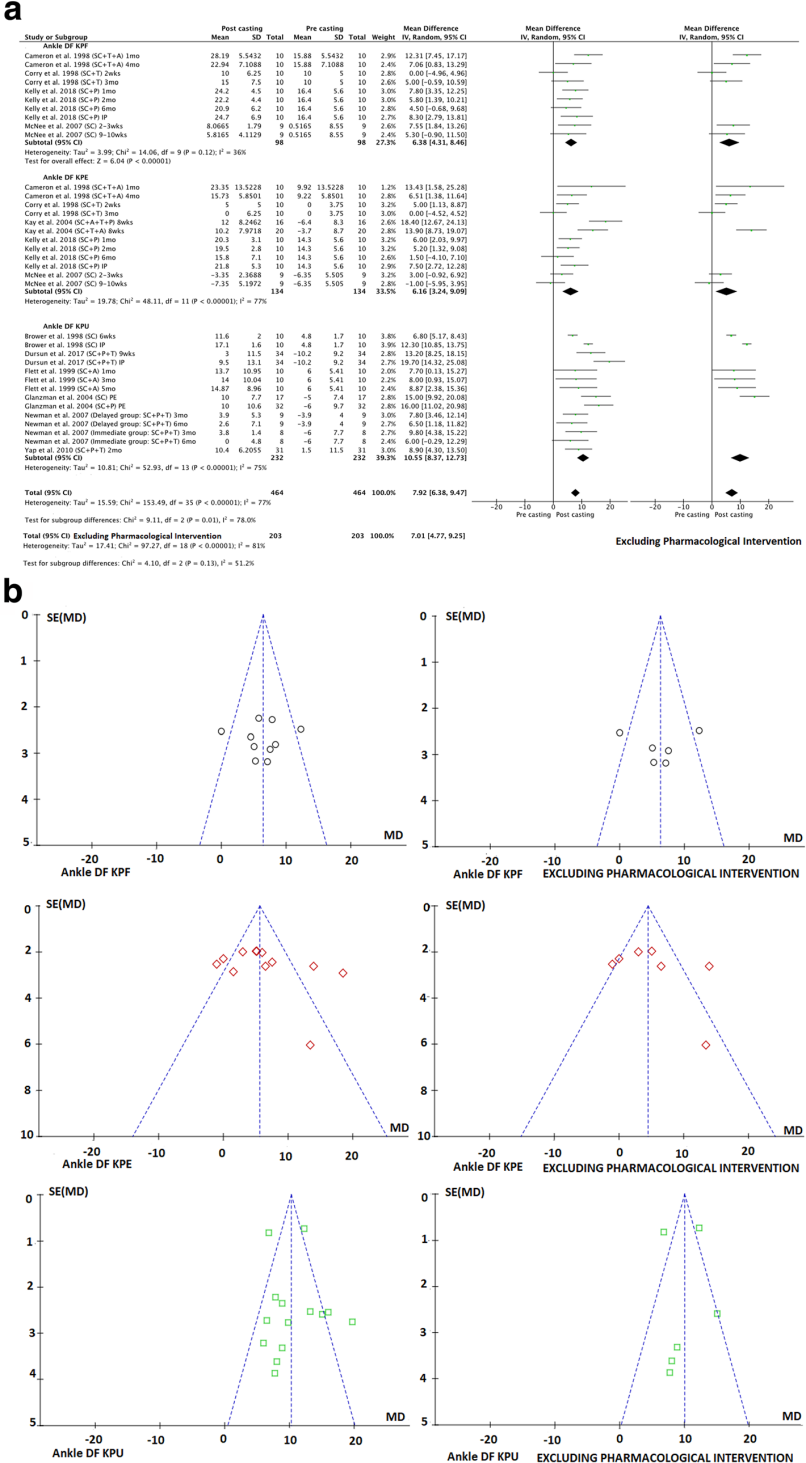
**a** Effects of serial casting on ankle dorsi-flexion for children with Cerebral Palsy. https://figshare.com/s/89970dbb9ab5ac3eb71a**b** Funnel plots for effects of serial casting on ankle dorsi-flexion passive range of motion for children with Cerebral Palsy https://figshare.com/s/5d1117d9b927018c0a41

**Fig. 3:**
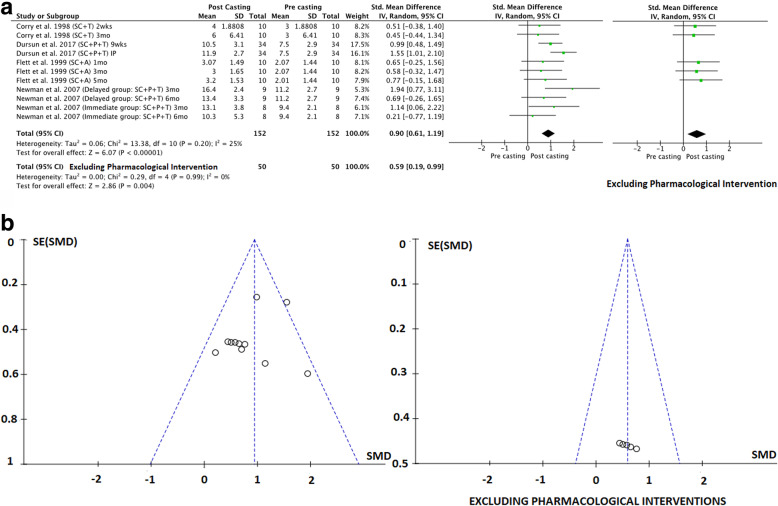
**a** Effects of serial casting on gait for children with Cerebral Palsy. https://figshare.com/s/bb37fa42c86e569fbca6**b**. Funnel plots for effects of serial casting on gait for children with Cerebral Palsy https://figshare.com/s/9bb27a5ca3ca5896db55

**Fig. 4:**
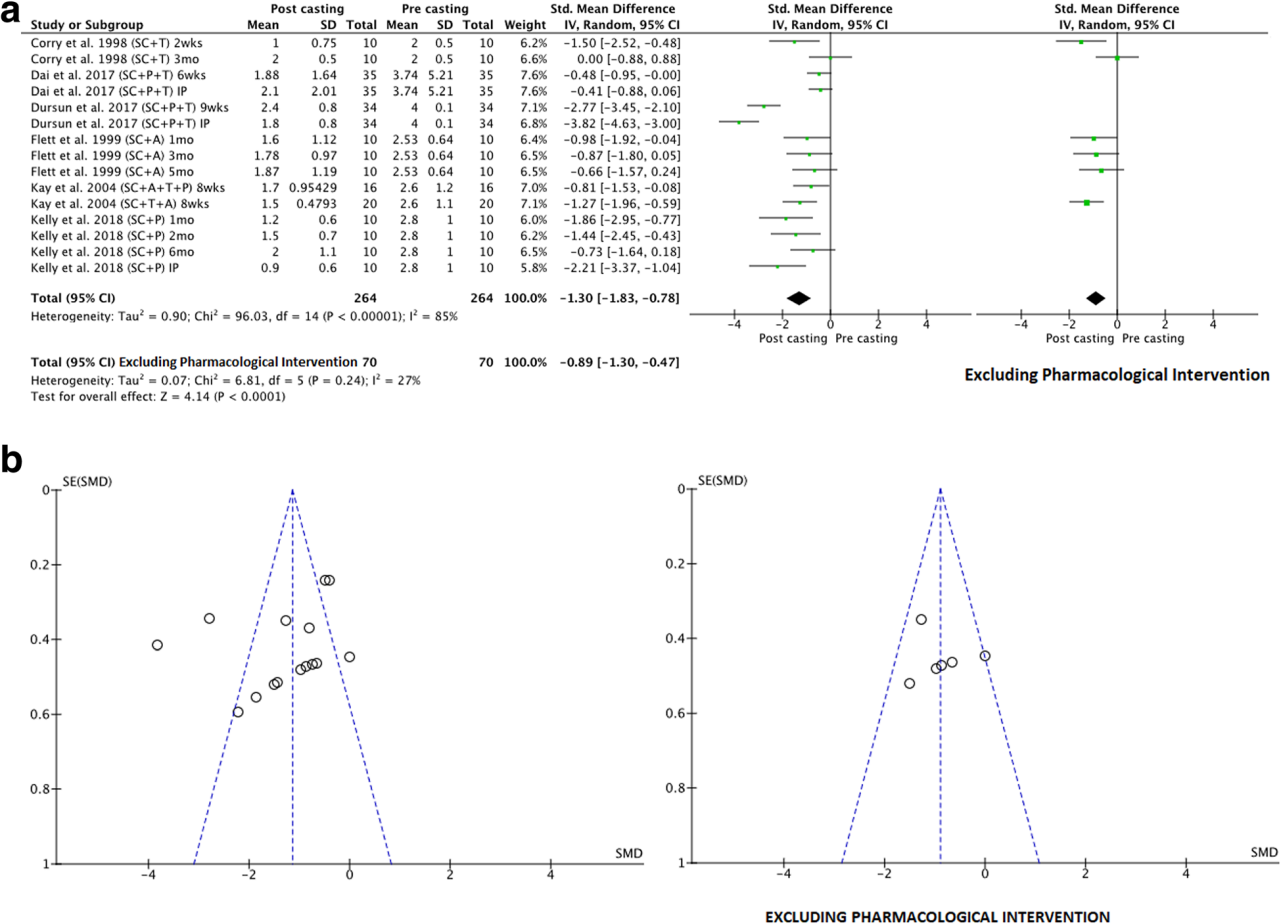
**a** Effects of serial casting on hypertonicity (MAS) for children with Cerebral Palsy. https://figshare.com/s/f92823032da7d3fed181**b** Funnel plots for effects of serial casting on hypertonicity (MAS) for children with Cerebral Palsy https://figshare.com/s/5faa9f389d0dedfd67b8

**Fig. 5:**
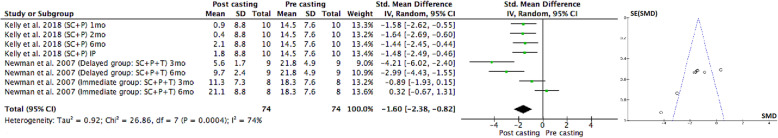
Effects of serial casting on spasticity for children with Cerebral Palsy with funnel plot https://figshare.com/s/d33f8e096262c8eab7fc

**Fig. 6:**
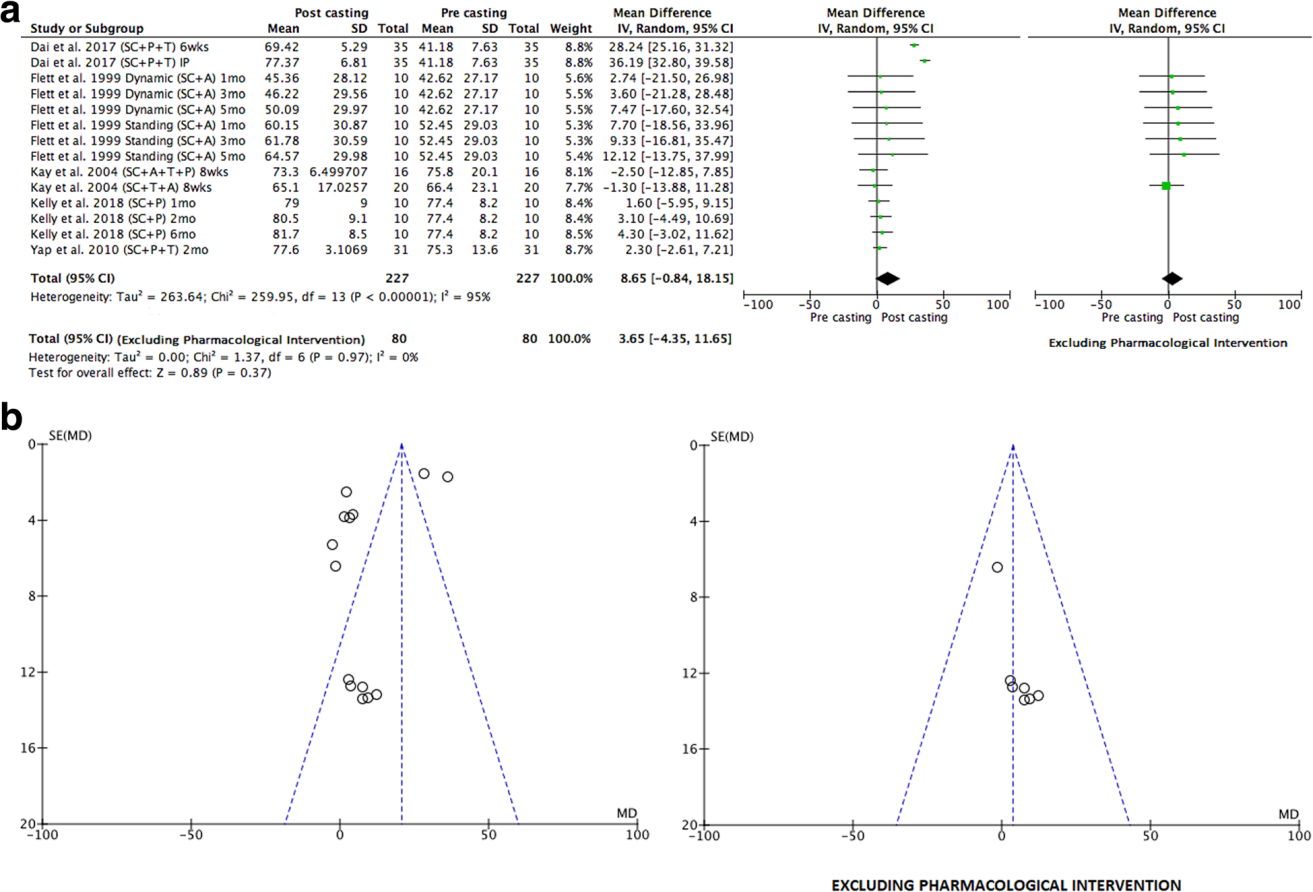
**a** Effects of serial casting on gross motor function for children with Cerebral Palsy. https://figshare.com/s/b88afff7075e75191432**b** Funnel plots for effects of serial casting on gross motor function for children with Cerebral Palsy https://figshare.com/s/112d0557375f01d43b4b

**Fig. 7:**
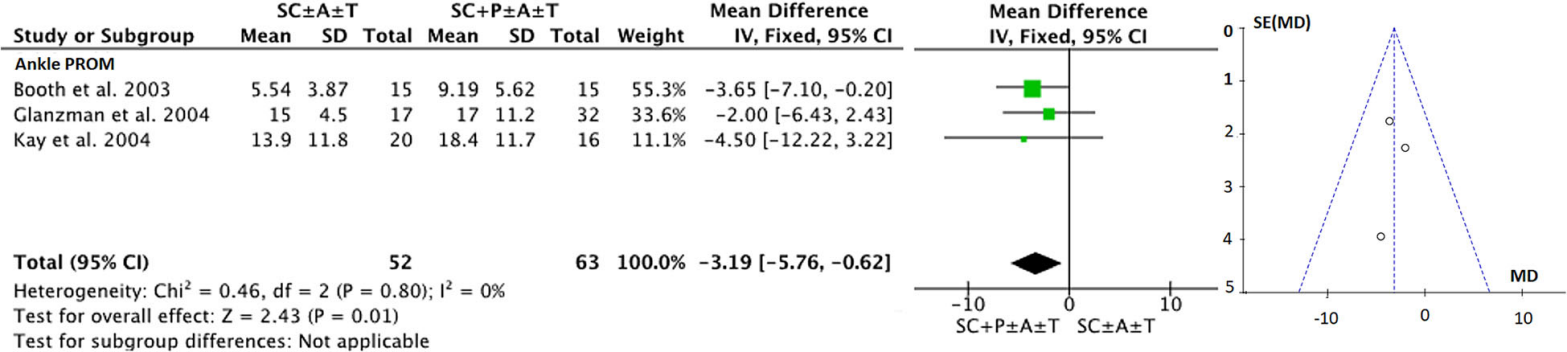
Effects of Serial Casting on Ankle Dorsi Flexion Range of Motion with versus without pharmacological intervention for children with Cerebral Palsy with funnel plot https://figshare.com/s/68959259873dad102b61

To address the first aim of this review, meta-analyses were conducted to determine the pre-to-post effects of serial casting on key outcome measures related to lower limb function that were included in the meta-synthesis. To address the second aim (investigating the longitudinal effects of serial casting), meta-analyses were conducted across four different timeframes post-completion of serial casting: i) Immediately post (IP), which included studies that completed their post-treatment assessment within seven days of final cast removal or reported only the peak effect (i.e. one post-casting measure); ii) Short-term (ST) > 7 days and < 3 months; iii) Mid-term (MT) between three months and > six months and; iv) Long-term (LT) > 6 months. A final meta-analysis was conducted addressing the third study aim; to contrast the effects of serial casting when coupled with pharmacological interventions aimed at impacting spasticity, hypertonicity or contracture (e.g. BTX-A, Baclofen), compared to serial casting without pharmacological intervention. For this analysis, only studies that investigated the results of serial casting compared to serial casting with pharmacological intervention were included. Interventions may have also used additional therapies (e.g. physiotherapy and orthoses). In addition to the timeframe coding listed above, an additional code has been used in the meta-analyses (Figs. 2, 3, 4, 5, 6 and 7) to document the combination of therapy interventions; i) Serial Casting (SC); ii) Pharmacological (P); Ankle Foot Orthosis / or similar lower limb orthoses (A); Therapy – Physical or Occupational (T).

## Results

### Literature search and selection

The database search identified 3219 citations, and after removing duplicates, 1921 articles were included for evaluation in this systematic review (see Fig. 1). After applying inclusion and exclusion criteria, 25 articles remained [11, 15, 16, 22, 28, 29, 31, 39, 44–60]. Collectively 414 children undergoing serial casting (mean age: 5.6 years) were included with 476 limbs assessed across all studies. Two of the studies included did not have a study population of children solely with CP. Brouwer et al. [44] and Brouwer et al. [29] included children that had idiopathic toe-walking; however, the pre- and post-casting results of the children with CP were able to be isolated for analysis. Glanzman et al. [16] included 11 participants that did not have CP; however, this was less than 20% of the total number of participants in that study and subsequently still met the inclusion criteria for the present review. Figure 1 illustrates the screening and selection process and final number of studies included as per the PRISMA protocol [36].

### Critical appraisal of methodological quality

The methodological quality score of the studies are presented in the data extraction table (see Table S1, Supplement File 1). Cohen’s κ was undertaken to determine inter-rater reliability with regards to reviewers’ judgment on scoring individual Downs and Black items for included studies. There was moderate agreement between the two reviewers, κ = 0.66, *p* < .0001. In total, there were differences in 17.6% of the scores for the Downs and Black [37]. Two reviewers (EB and MM) resolved 15.8% of the differences in scoring, and a third reviewer (NM) assisted in resolving 1.8% of the scoring.

It was identified using the Downs and Black critical appraisal tool that no studies represented the entire source population (i.e. all children diagnosed with CP) from which they were recruited (item #11) and to the same manner, no subjects who participated, represented the entire population from which they were recruited (item #12). Nine studies were deemed to have poor methodological quality [29, 47, 50, 51, 54–57, 59], eleven studies were deemed to have fair methodological quality [15, 16, 22, 28, 39, 44–46, 49, 53, 58] and five studies were found to have good methodological quality [11, 31, 48, 52, 60]. When critically appraising the internal validity of studies, items relevant to the recruitment period of participants (item #22), blinding to randomized assignment (item #24) and adjustment for confounding variables (item #25) were all major contributors impacting the methodological quality studies included in this systematic review. Furthermore, a lack of power analysis was noted in most of studies with only 20.0% of studies reporting power (item #27). All articles scored ‘0’ for item #14 and only six (25%) articles scored ‘1’ for item #15. These two questions were related to blinding subjects and blinding the individuals measuring the outcomes respectively, and this contributed to the reduced overall internal validity of the studies included in this systematic review. Additionally, there were many studies that did not report losses to follow up, further impacting the quality rating scores of those studies. See Table S1 (Supplement File 1) for a detailed summary of all studies included in this review and their critical appraisal scores.

Although sixteen studies with fair to good methodological quality explored the effects of serial casting on lower limb function, results from a number of investigations included in Table S1 did not meet the pre-defined criteria of a minimum of five investigations from fair to good methodological studies, and for this reason were not included in the meta-synthesis (Tables 3 and 4). Effects of serial casting on particular outcomes were not included in meta-synthesis when the associated studies did not meet the threshold for methodological quality or when the required number of investigations for a particular outcome to be included was not met, and consequently the outcome measures that were not included in meta-synthesis comprised multiple body structure or function measures as well as activity and participation level outcomes. The body structure and function measures that were not included in meta-synthesis were: knee extension PROM [50, 52, 55, 56], hip abduction PROM [51], Thomas Test [51], knee PROM (popliteal angle) [50, 51], ankle dorsiflexion AROM [58], knee extension AROM [56], ankle angle at initial foot contact [46, 49], Modified Ashworth Scale (MAS) assessing the hamstrings [52, 56], Ashworth Scale (not modified) [46], velocity of passive stretch [29, 44], Tardieu Scale (not modified) [11], Modified Tardieu Scale R1 angle ankle DF [54], dynamic assessment of ROM (DROM) [50], reflex excitability [29, 44], isometric plantar flexion (PF) strength [44] and Physiological Cost Index (PCI) [45]. Activity or participation level measures that were not included were: gait velocity [45], gait-related cadence [39, 52], WeeFim mobility scale [58], the Berg Balance Test [56] and Paediatric Evaluation of Disability Inventory (PEDI) [52]. The remaining measures included for meta-synthesis have been grouped according to clinical utility themes and are presented as: i) Lower Limb Range of Motion; ii) Functional Gait Measures; iii) Neurological Assessments and; iv) Gross Motor Function.

### Populations and interventions identified in studies with fair to good methodological quality

The 16 studies with fair to good methodological quality had relatively small study cohorts, ranging from nine to 80 participants, with a mean of 28 participants per study. Four hundred and forty participants undergoing serial casting were included across these studies. The age of participants ranged from two to 14 years with a mean age of 5.9 years. The materials used, and the positioning and handling of patients was varied across studies. Two studies [15, 46] did not state their casting protocol with enough detail to replicate in future studies, six studies reported the use of fiberglass [28, 31, 39, 52, 53, 60], while two reported using Plaster of Paris (POP) casting materials [15, 48]. Two studies [52, 60] published protocols to specify patient positioning in supine during application of casting, while five other studies reported positioning in prone [16, 28, 39, 45, 53]. Six studies specified that the knee was flexed to 90 degrees [16, 29, 39, 52, 53, 60] during the application of casting and four reported that the sub-talar joint was positioned into neutral with the forefoot aligned to the hind-foot [29, 45, 52, 60]. A plaster boot or walking shoe was provided for subjects in seven studies [31, 39, 45, 52, 53, 58, 60]. Serial casting duration and frequency were also varied across the 16 fair to good studies. Casts were applied for durations varying between 72 h to four weeks and between one to four casts were used across the serial casting protocols. All studies intended to use two or more casts during the protocol if required. Assessment timeframes for all reported outcome measures ranged widely from immediately post-completion of serial casting through to more than 12 months post-final cast removal. Thirteen of the studies with fair to good methodological quality explored the use of BTX-A as a pharmacological intervention to improve outcomes relevant to lower limb structure, function or activity [11, 15, 16, 22, 28, 31, 46, 48, 49, 52, 53, 58, 60]. Whilst this review intended to report the outcomes of studies using any additional pharmacological intervention to address spasticity, when coupled with serial casting, no pharmacological intervention, other than BTX-A, was explored in the included studies with fair to good methodological quality. For this reason, the remainder of the results will refer to BTX-A when appropriate for reporting on pharmacological interventions.

### Aims 1 and 2: overall and longitudinal effectiveness of serial casting as a therapeutic intervention in isolation or in combination with other therapies (which may include pharmacological intervention)

The primary outcomes that met the threshold for inclusion in the meta-synthesis were relevant to: i) Lower limb range of motion; ii) Functional gait; iii) Neurological measures and; iv) Gross motor function (see Tables 3 and 4). The results from studies with fair to good methodological quality are narratively synthesized below.

#### Lower limb range of motion

Fourteen out of the sixteen studies of fair to good methodological quality reported the effects of serial casting on PROM at the ankle [11, 15, 16, 22, 28, 31, 39, 44–46, 52, 53, 58, 60], at both baseline and post-casting intervention. Within these 14 studies, there were 42 individual investigations exploring the effects of serial casting on lower limb PROM. For ankle DF PROM, there were 18 investigations [11, 15, 16, 22, 28, 44, 53, 58] where the knee position was unspecified (KPU), 11 investigations [39, 45, 46, 52, 60] where the knee position flexed (KPF) and 13 investigations [31, 39, 45, 46, 52, 60] with the knee position extended (KPE) during assessment. The summary coding for the meta-synthesis (see Tables 3 and 4) revealed an overall positive/desirable effect when using serial casting to improve ankle DF PROM in children with CP. This desirable effect was maintained when investigations using BTX-A were removed (see Table 4).

The results of a meta-analysis of multiple investigations across 11 studies [11, 16, 22, 31, 39, 44–46, 53, 58, 60] with fair to good methodological quality that reported ankle DF PROM outcomes are presented in Fig. 2a. Studies were grouped according to knee position: flexed (KPF), extended (KPE), or unspecified (KPU). Three articles measuring this outcome were not included in the meta-analysis as they did not report baseline data [15, 28, 52]. The overall combined results demonstrated substantial heterogeneity (77%) when the studies were inclusive of pharmacological intervention, which in all cases was BTX-A and when meta-analysis was independent of pharmacological intervention substantial heterogeneity remained (81%), consequently, it was not appropriate to calculate a pooled effect estimate to assess the overall effect of serial casting on ankle DF PROM (see Fig. 2a). Additionally, funnel plots represented in Fig. 2b suggest that publication bias may be present for studies investigating the effects of serial casting on ankle DF PROM with KPE, although publication bias cannot be confirmed due to the high levels of heterogeneity across the studies. However, serial casting was shown via meta-analysis to significantly improve Ankle DF PROM when the knee position was unspecified (KPU) in the immediate (IP) and short term (ST) (independent of pharmacological intervention) and in the mid-term (MT) (inclusive of pharmacological intervention – which in all cases was BTX-A) (see Table 5). When the knee position was flexed during measurement (KPF), serial casting had a significant desirable effect on Ankle DF PROM in the mid-term (MT) with BTX-A included and without pharmacological intervention (see Table 5). There were no investigations exploring the effects of serial casting on DF PROM at long-term follow up beyond 6 months.

**Table 5:** Longitudinal and Collective Effects of Serial Casting on the Lower Limb for Children with Cerebral Palsy

Outcome / Measure		Effect of Serial Casting									
		Inclusive of additional therapeutic interventions					Studies with pharmacological intervention (in all cases this was BTX-A) were removed from meta-analyses				
Ankle DF KPU	Timelines	Studies	Mean difference (MD or *SMD)	Confidence Interval	Hetero-geneity	Test for Overall Effect Z Score (*p*-value)	Studies	Mean difference (MD or *SMD)	Confidence Interval	Hetero-geneity	Test for Overall Effect Z Score (*p*-value)
	IP	44, 11, 22, 16	15.15	[11.74, 18.56]	I^2^ = 66%	NC	44, 16	12.51	[11.10, 13.92]	I^2^ = 0%	Z = 17.35 (*p* < 0.00001)
	ST	44, 11, 22, 58	8.74	[5.76, 11.73]	I^2^ = 51%	NC	44, 22	6.84	[5.25, 8.43]	I^2^ = 0%	Z = 8.43 (*p* < 0.00001)
	MT	22, 53	8.54	[5.78, 11.31]	I^2^ = 0%	Z = 6.06 (*p* < 0.00001)	22	8.47	[3.69, 13.25]	I^2^ = 0%	Z = 3.47 (*p* = 0.0005)
	LT	53	6.29	[2.23, 10.35]	I^2^ = 0%	Z = 3.03 (*p* = 0.002)	–	–	–	–	–
Ankle DF KPF	IP	–	–	–	–	–	–	–	–	–	–
	ST	45,46, 60, 39	6.50	[3.21, 9.79]	I^2^ = 61%	NC	45, 46, 39	6.31	[0.84, 11.79]	I^2^ = 76%	NC
	MT	–	–	–	–	–	45, 46	5.92	[1.76, 10.08]	I^2^ = 0%	Z = 2.79 (*p* = 0.005)
	LT	–	–	–	–	–	–	–	–	–	–
Ankle DF KPE	IP	–	–	–	–	–	–	–	–	–	–
	ST	45, 46, 31, 60, 39	7.42	[3.44, 11.40]	I^2^ = 82%	NC	45, 46, 31, 39	6.05	[0.93, 11.18]	I^2^ = 80%	NC
	MT	45, 46	3.14	[−3.24, 9.51]	I^2^ = 71%	NC	45, 46	3.14	[−3.24, 9.51]	I^2^ = 71%	NC
	LT	–	–	–	–	–	–	–	–	–	–
Functional Gait (Objective Standardised Measures)	IP	–	–	–	–	–	–	–	–	–	–
	ST	46, 11, 22	* 0.83	[0.43, 1.23]	I^2^ = 0%	Z = 4.11 (*p* < 0.0001)	46, 22	* 0.58	[−0.06, 1.22]	I^2^ = 0%	Z = 1.79 (*p* = 0.07)
	MT	46, 22, 53	* 0.88	[0.41, 1.36]	I^2^ = 16%	Z = 3.63 (*p* = 0.0003)	46, 22	* 0.59	[0.07, 1.11]	I^2^ = 0%	Z = 2.24 (*p* = 0.03)
	LT	–	–	–	–	–	–	–	–	–	–
Neuro – Hypertonicity (MAS) PF	IP	48, 11, 60	* -2.13	[−4.43, 0.18]	I^2^ = 96%	NC	–	–	–	–	–
	ST	46, 48, 11, 22, 31, 60	* -1.37	[−1.97, −0.77]	I^2^ = 79%	NC	46, 22, 31	* -1.24	[−1.73, −0.76]	I^2^ = 0%	Z = 5.01 (*p* < 0.00001)
	MT	–	–	–	–	–	46, 22	* -0.49	[− 1.02, 0.03]	I^2^ = 0%	Z = 1.86 (*p* = 0.06)
	LT	–	–	–	–	–	–	–	–	–	–
Neuro – Spasticity (MTS) (R2-R1)	IP	–	–	–	–	–	–	–	–	–	–
	ST	60	* -1.61	[−2.35, − 0.88]	I^2^ = 0%	Z = 4.30 (p < 0.0001)	–	–	–	–	–
	MT	53	* -2.46	[−5.71, 0.79]	I^2^ = 90%	NC	–	–	–	–	–
	LT	60, 53	* -1.31	[−3.09, 0.47]	I^2^ = 87%	NC	–	–	–	–	–
GMFM	IP	–	–	–	–	–	–	–	–	–	–
	ST	48, 22, 31, 60, 58	5.49	[−6.46, 17.45]	I^2^ = 95%	NC	22, 31	0.80	[−9.47, 11.08]	I^2^ = 0%	Z = 0.15 (*p* = 0.88)
	MT	–	–	–	–	–	22	8.03	[−4.71, 20.76]	I^2^ = 0%	Z = 1.23 (*p* = 0.22)
	LT	–	–	–	–	–	–	–	–	–	–

*IP* Immediately post serial casting (within 7 days of final cast removal), *ST* Short-term post serial casting (> 7 days and < 3 months), *MT* Mid-term post serial casting (between 3 months and < 6 months), *LT* Long-term post serial casting (> 6 months)

Ankle DF (Dorsi Flexion) KPU (Knee Position Unspecified), KPF (Knee Position Flexed), KPE (Knee Position Extended). GMFM (Gross Motor Function Measure)

*MD* Mean Difference, *SMD Standardised Mean Difference (used when outcome is not measured in consistent units or with consistent tools)

Significance value for Test of Overall Effect (Z) set at *p* < 0.05

*NC* Not calculated (when heterogeneity I^2^ was either substantial (50–90%) or considerable (> 90%) the pooled effect estimate was not calculated

– represents insufficient data to undertake the meta-analysis

Note: where one study is listed individually, a minimum of two investigations from that study were utilised for the meta-analysis

#### Functional gait measures

Ten studies of fair to good methodological quality reported data on one or more of the following outcomes related to functional gait: velocity [39, 45, 52, 60], stride length [39, 45, 52, 60], Observational Gait Scale (OGS) [11, 53] and the Physician’s Rating Scale (PRS) [22, 46, 58]. Meta-syntheses revealed that serial casting (inclusive of additional therapeutic interventions) had no significant effect on velocity and stride length (see Table 3). The effect of serial casting (without pharmacological intervention) on velocity or stride length was questionable (see Table 4) due to the minimum number of investigations not meeting the threshold for meta-synthesis. Results of the meta-synthesis suggest that serial casting (both with BTX-A and without pharmacological intervention) results in a desirable outcome on functional gait when measured with the Physicians Rating Scale (PRS). There were insufficient studies reporting cadence or ankle angle at initial contact to undertake a meta-synthesis or meta-analysis. Results from a meta-analysis comparing baseline and post-casting data for Observational Gait Scale (OGS) and PRS were presented together as objective standardized gait measures, and are reported in Fig. 3a, demonstrating that serial casting (inclusive of additional therapeutic interventions) has an overall positive effect on functional gait and although slightly less effective the positive effect remained when studies using additional pharmacological intervention were removed from the analysis (see Fig. 3a). Funnel plots in Fig. 3b, revealed that publication bias did not impact these results. Stride length and velocity were not included in the meta-analysis as their summary coding from the meta-synthesis demonstrated a non-significant effect from serial casting (see Table 3).

When exploring the longitudinal effects of serial casting on functional gait the results of meta-analyses identified a positive significant effect of serial casting (inclusive of additional therapeutic interventions) in the short and mid-term timeframes. These effects remained positive in the mid-term but not in the short term when studies using additional pharmacological intervention (which in all cases was BTX-A) were removed from the analysis (see Table 5). Minimal heterogeneity was noted across the studies. There were insufficient data to explore, via meta-analyses, the immediate post effects of serial casting on functional gait outcomes. Based on two investigations from one study [53], serial casting (inclusive of additional therapeutic interventions) did not appear to have a significant long-term effect on functional gait outcomes (see Table 5).

#### Neurological assessments

Eight studies of fair to good methodological quality reported data from neurological assessment measures including: The Ashworth/Modified Ashworth Scale (MAS) assessing hypertonicity in both hamstring [52] and ankle plantar-flexor muscle groups [11, 22, 31, 46, 48, 52, 58, 60], the Tardieu/Modified Tardieu Scale (MTS) indicating R1 (spastic catch) angle for knee extension [52], or R2-R1 difference (spasticity) for ankle plantar-flexors [52, 53, 60]. There were sufficient investigations to determine the effect of serial casting on only two neurological outcomes via meta-synthesis. These were the MAS for ankle plantar-flexors (n/*N* = 82.35%), and MTS (R1, R2-R1) (n/*N* = 70.00%), suggesting a desirable effect of serial casting (inclusive of additional therapeutic interventions) for reducing hypertonicity (see Table 3) and a questionable effect of serial casting for reducing (MAS) when interventions were not inclusive of pharmacological input (due to a limited number of investigations – see Table 4). A meta-analysis was conducted with studies that measured the effects of serial casting on hypertonicity using the MAS. The overall combined results inclusive of pharmacological intervention (which in all cases was BTX-A) demonstrated substantial heterogeneity (85%) across the studies, consequently, it was not appropriate to calculate a pooled effect estimate (see Fig. 4a). However, when meta-analysis was undertaken independent of studies using pharmacological intervention the overall effect of serial casting on hypertonicity of the ankle plantar flexors was shown to be significantly desirable (see Fig. 4a) and this significant desirable effect occurs mostly in the short term (ST) (see Table 5). The funnel plot for the meta-analysis in Fig. 4b, revealed that there was no evidence of publication bias impacting the results when studies using pharmacological interventions were removed. There was no data to examine the effects of serial casting on hypertonicity in the long term.

A meta-analysis was also conducted with studies that measured the effects of serial casting (inclusive of additional therapeutic interventions) on spasticity using the MTS. The overall combined results inclusive of BTX-A demonstrated substantial heterogeneity (74%) across the studies, consequently, it was not appropriate to calculate a pooled effect estimate (see Fig. 5) and publication bias could not be accurately assessed. When meta-analyses were undertaken to explore the effects of serial casting on spasticity, a small but significant effect was demonstrated in the short-term (ST) when BTX-A injections to the gastrocnemius were included in the protocol (see Table 5) however this was not maintained in the mid (MT) or long-term (LT). Insufficient data was available to undertake a meta-analysis to examine the effects of serial casting on lower limb spasticity independent of pharmacological intervention.

#### Gross motor function

Six studies [22, 31, 48, 52, 58, 60] of fair to good methodological quality reported the effects of lower limb serial casting on gross motor proficiency, using the Gross Motor Function Measure (GMFM). The meta-synthesis (Table 3) revealed that serial casting of the lower limb (inclusive of additional therapeutic interventions) had an overall desirable effect on gross motor function and this desirable effect remained when investigations inclusive of pharmacological intervention were not included. However, results from a meta-analysis comparing baseline to post-casting GMFM scores (inclusive of additional therapeutic interventions) demonstrated considerable heterogeneity (95%) across the studies, consequently, it was not appropriate to calculate a pooled effect estimate (see Fig. 6a) and publication bias could not be accurately assessed from the funnel plots (see Fig. 6b). When studies including pharmacological interventions (which in all cases was BTX-A) were removed from the meta-analysis results showed that serial casting did not have a significant effect on gross motor function (GMFM) (see Fig. 6a). When exploring the longitudinal effects of lower limb serial casting (independent of pharmacological intervention) on gross motor proficiency, meta-analyses revealed no significant changes in GMFM scores between baseline and follow-up in the short-term (ST) and mid-term (MT), post serial casting (see Table 5). There were insufficient published investigations to determine the immediate effects of serial casting on gross motor function and there were no studies that explored the long-term effects of serial casting on gross motor function for children with CP (see Table 5).

### Aim 3: determining if the addition of pharmacological intervention enhances the effects of serial casting

Only three studies [16, 28, 31] assessed the effects of serial casting (SC ± A ± T) compared to the effects of serial casting in combination with pharmacological intervention (SC ± P ± A ± T). The pharmacological intervention in all three studies was identified as BTX-A. One study was shown to have good methodological quality [31] and two had fair methodological quality [16, 28]. From these studies, the only common outcome measure was ankle DF PROM which was collectively measured with knee in an extended (KPE), or unspecified (KPU) position. Figure 7 demonstrates a small but positive overall effect favouring serial casting with the addition of BTX-A by just over three degrees. Heterogeneity was considered minimal suggesting the three studies were similar in terms of their findings and publication bias did not appear to be impacting the results, however the small number of studies limit the accuracy of using funnel plots to make this assessment. As the study by Booth et al. [28] provided the average change per week rather than the absolute pre-post casting change in DF PROM, a sensitivity analysis was undertaken to explore the effect of serial casting when this study [28] was removed. The results of the sensitivity analysis showed the results were slightly modified (MD − 2.62 degrees; 95% CI − 6.46 to 1.22; *P* = 0.18; I^2^ - = 0%) and whilst the addition of BTX-A still achieved greater ankle DF PROM, the difference between the two intervention methods became non-significant when the study by Booth (2003) was removed.

### Adverse events from intervention

All adverse events from interventions (when reported) were documented in Table S1. Supplement File 1 (Data Extraction Table). Adverse events documented when BTX-A was utilised across all studies included: Calf pain (*n* = 3), with Dai (2007) [48] documenting ‘most patients’ suffered from calf pain; Excessive weakness (*n* = 1); Increased micturition and faecal soiling for 1-month post injection (n = 1) and three studies [11, 16, 22] stated that no patients displayed adverse events when their protocol included BTX-A. Adverse events noted in studies utilising serial casting (independent of pharmacological intervention) across all studies included: Foot pain (*n* = 4); Calf pain (*n* = 2); Skin inflammation (*n* = 3); Pressure sores – grade 1–2 (*n* = 5) and; Lower limb atrophy (*n* = 3). A small number of concerns were also noted with dissatisfaction with the procedure of serial casting (*n* = 3) and difficulty with home-care bathing (*n* = 4) were also noted across the studies collectively. Adverse events from utilising combined BTX-A and casting were noted in two studies and included: Lower limb weakness (*n* = 12); Pain (*n* = 3); Lower limb atrophy (*n* = 12); Temporary incontinence (*n* = 3); Constipation (*n* = 1); Pressure sores (*n* = 1) and; Tendonitis (*n* = 1). Nine of the included studies [28, 39, 44, 45, 50, 54, 56–58] did not report adverse effects of the intervention in their manuscripts and a further four studies [29, 31, 52, 60] stated that there were ‘not adverse events’ during their interventions.

## Discussion

The overall objective of this systematic review was to identify the efficacy of serial casting as an intervention in the management of lower limb dysfunction in children with Cerebral Palsy. Other systematic reviews have addressed the utility of serial casting as an adjunct to pharmacological intervention [32, 33] or are now considered to be out of date [17, 21, 25]. No recent publications have summarized findings solely according to higher methodological quality studies to determine the effect of serial casting on lower limb function, nor have recent publications provided a meta-analysis of findings relevant to the study aims to allow more robust conclusions. The results of this systematic review with meta-analyses suggest that serial casting (independent of BTX-A) can have significant desirable effects on i) increasing ankle DF PROM in the immediate (IP) to mid-term (MT); ii) reducing hypertonicity in the short-term (ST) and; iii) improving functional gait in the mid-term (MT). Furthermore, the findings of this review indicate that the effects of serial casting on ankle DF PROM are further and significantly enhanced by the combination of BTX-A, however the clinical significance of the magnitude of this effect remains unclear. These findings support the use of serial casting in clinical practice to manage impairments at the body function and structure level to improve lower limb activities such as gait.

Relevant to the first aim, the strongest findings were in the immediate (IP) and short-term (ST) periods post casting, when the knee position was unspecified (KPU) for DF PROM measurement, which made it difficult to differentiate the most implicated muscles involved in the overall effect. However, serial casting appeared to have the strongest effect on the soleus muscles of the triceps-surae three to six months after serial casting. This may be the result of the gastrocnemius muscles shortening quicker than the soleus muscles after earlier gains from serial casting. However, in order to truly understand what components of casting are causing the most effective outcomes, it is recommended that future studies include more detailed reporting of their casting protocols (i.e. casting duration and position, subject positioning, weight bearing status, casting material, as well as adjunct therapy before, during and after serial casting) and measurement protocols, to enable better identification of possible contributors for the effects of serial casting. For example, if a below-knee cast is applied, with the intent to lengthen the gastrocnemius, one may clinically reason that the footplate of the cast needs to end distal to the first toe, with the footplate of the cast having a flat sole, without the addition of an inserted rocker, and that a regular exercise program utilising full knee extension in standing and walking activities should be prescribed. If the details of the casting protocol were consistently described, it may be possible for future reviews to identify which components are having the most effect on the studied outcomes.

When used in clinical practice, the main aim of serial casting is to increase PROM around the ankle to eventually achieve adequate AROM at the ankle to reach heel strike at first foot contact, to consequently improve functional gait parameters and other age-specific functional outcomes. Data synthesis (see Tables 3 and 4), identified that serial casting overall did not have a significant effect on walking velocity or stride length. However, it is important to note that for walking velocity, only two studies reported significant effects, one of which [52] claimed that that serial casting had a significant undesirable effect on walking velocity and stride length immediately post-cast removal (one week after final cast removal). However, statistical results were not reported for stride length and this study used serial casting after BTX-A injections. This undesirable effect is most likely attributed to the weakening impact of BTX-A and the negative implications that prolonged immobilization has on muscular strength, and this hypothesis is supported by the increased adverse effects for children when serial casting and BTX-A were combined. This negative effect has been described previously in the empirical literature [61], where ankle plantar-flexion torque, fatigue resistance and functional recovery following casting for ankle fracture were examined. Reduced ability to generate plantar-flexor muscle force has direct implications during the late stages of stance phase, which can ultimately influence stride length and gait velocity [62]. This undesirable effect indicates a need for intermittent serial casting with lower limb strengthening incorporated in-between cast applications, as opposed to continuous casts over prolonged periods. The recommendations by Novak and colleagues in 2013 [17], regarding lower limb strength training and electrical muscle stimulation, appear critical for enhancing a child’s strength and therefore function, post- and perhaps during serial casting episodes, especially when using prolonged serial casting protocols. Most studies with intermittent casting protocols were excluded from this systematic review as they failed to meet our pre-determined definition of serial casting. Results pertaining to muscle strength post-casting were not reported in this review as there were not sufficient studies investigating this outcome in the included literature. Further investigation into the effects of combining serial casting with brief (e.g. 1–2 days) non-casting periods of age-appropriate strengthening activities is therefore warranted.

Another desirable effect of serial casting identified in this review was the reduced influence of hypertonicity on muscles around the ankle joint, in the short-term (ST) post-cast removal. The authors of this review feel that these benefits may have translated into the improvement in functional gait described in some studies [22, 53], with improved ankle DF PROM allowing for improved clearance of the foot in the mid-swing phase of a limb implicated with hypertonicity. Important to addressing the aims of this study, functional gait improvements (using objective standardised measures) were observed in studies that used serial casting without pharmacological intervention (see Table 4) although meta-analyses did not demonstrate a carry-over effect with gross motor skills as significant changes in gross motor function measure scores were not observed. An improved ability to reach heel strike may have accounted for improved balance, which is reflected in the improved functional gait outcomes reported in this review. The decrease in hypertonicity in the lower limb post-casting reported in studies assessing hypertonicity using the MAS [11, 22, 31, 48, 52, 58] may also have positively impacted functional gait for similar reasons. Whilst the MAS is a commonly used measure in clinical practice, future research could use more objective measures (e.g. electromyography) to quantify the responses of serial casting on hypertonicity levels, which may allow for more definite assessments of the relationship between decreased levels of hypertonicity and improved gait and / or gross motor outcomes.

In addressing the second aim of this review; the effects of serial casting longitudinally, the authors identified that there were no fair or good quality studies investigating the long-term effects of serial casting (i.e. greater than six months) independent of pharmacological intervention, on lower limb function for ankle DF PROM, hypertonicity (MAS), spasticity (MTS), gait (OGS) or gross motor function (GMFM). It is therefore recommended that future longitudinal studies are conducted to determine the efficacy and sustainability of serial casting as an intervention for long-term management of lower limb dysfunction in children with CP. The findings from future longitudinal studies, particularly if compared to long-term effects of additional pharmacological interventions, would further assist parents/carers in their decision-making regarding preferred methods of intervention. These options should then be discussed with children and their carers using shared decision-making models for treatment planning [63].

The findings related to the third aim of this review suggest that the addition of pharmacological intervention to serial casting results in slightly yet significantly better ankle DF PROM; however, these findings should be interpreted with caution. An additional two-to-three degrees of ankle DF PROM may translate to be functionally beneficial if the ankle DF range of an individual is within a few (two to three) degrees of achieving neutral ankle DF. However, this may not be functionally beneficial if the individual has either i) already achieved neutral ankle DF, or, ii) is substantially greater than three degrees away from achieving neutral ankle DF from an originally plantarflexed position. During human gait, critical foot clearance is influenced by slight movements at the ankle joint. A study undertaken by Moosabhoy and colleagues in 2006 [64], suggested that the point of critical toe clearance occurs when the ankle is slightly dorsiflexed (at approximately one degree past neutral). Therefore, if the addition of two-to-three degrees ankle DF from the pharmacological intervention (i.e. BTX-A) achieves range through the point of critical toe clearance, then it is likely to be of clinical value above and beyond serial casting alone. Importantly, our sensitivity analysis showed that the difference in DF PROM between serial casting and serial casting with pharmacological intervention (which in all cases was BTX-A) became non-significant when one study [28] that used alternative measurement options was removed from the meta-analysis. This suggests that the findings rest on limited data and further high quality randomized controlled trials exploring the differences between serial casting and serial casting with BTX-A or other pharmacological interventions are needed. Further, only one of the three studies clearly identified the knee position as being extended during measurement of ankle PROM and whilst the other two studies implied that the knee was extended, implicating the effect in the gastrocnemius muscles, there was a lack of clarity from authors of the included studies for reporting the measurement protocols. In view of our findings, it is important for clinicians to take into consideration that certain pharmacological interventions such as BTX-A are neurotoxins and whilst medically controlled they are considered safe for use with children, however, are generally more invasive with potentially higher risks [65]. Consequently, it is imperative that clinical reasoning and specific goal-setting related to lower limb function is incorporated in the family-centered decision-making process prior to recommending the addition of BTX-A to serial casting, on the basis that it is likely to result in only slightly better ankle DF PROM (see Fig. 7) with potentially more adverse effects.

In undertaking this systematic review, the authors identified several limitations in the published empirical literature. Many of the articles included had poor methodological quality, which was commonly attributed to a lack of detailed reporting of either the methods and/or results of the study. For example, many studies failed to report actual probability values, did not use appropriate statistics, or did not report statistical tools used. Several studies used the ANOVA statistical method of analysis without reporting a post-hoc analysis, preventing the identification of the point of significant difference from baseline to each post-treatment measure. This lack of detailed reporting also limited the ability to include such studies in the meta-analyses for this systematic review. Consequently, the authors of the present review needed to use estimation calculations or to exclude data from meta-analyses, which limited the ability to draw firm conclusions regarding the aims of this review and may have contributed to the substantial heterogeneity observed in many of the meta-analyses. In future research, it is recommended that authors report all statistics thoroughly, particularly mean values with standard deviations for transparency of findings and or publish their complete set of de-identified data. This will enable more sound analyses to be made in future systematic reviews and consequently firmer conclusions can be made.

The inconsistency of the data from the variety of assessment methods and outcome measures used, made it difficult to synthesize and quantify the magnitude of the reported serial casting effects. Additionally, the method of cast application was varied and on many occasions was not reported at all, making it challenging to analyze and quantify the potential impact of different serial casting protocols on lower limb function. Factors such as the duration of serial casting, the goal of serial casting, materials utilized, and different combinations of adjunct therapy varied immensely across the included studies and were also likely contributors to the large degree of heterogeneity, commonly prohibiting pooled effect estimates being calculated. The authors of this review recommend that future research relevant to this topic consider using casting protocols that are already prevalent in the literature or report their methodology in a detailed manner which would permit replication of methods, to allow the effects of serial casting to be thoroughly explored in future systematic reviews. The authors of this review acknowledge that to do this, journal publishers need to allow for extended word counts in presenting the methods of studies undertaken. Furthermore, this review revealed large degrees of heterogeneity for a number of analyses, and this was mostly related to the inconsistency of casting protocols, age populations and GMFCS classifications. Blackmore and colleagues in 2007 [21], reported a similar issue, stating that studies in their review had small sample sizes and lacked randomization and blinding, making it challenging to identify the true effects of serial casting as an intervention. To reduce the impact of these limitations, future studies should report detailed assessment and intervention protocols and procedures. Additionally, randomized controlled trials contrasting the effects of serial casting versus serial casting with BTX-A or other pharmacological interventions would also be useful for therapists to assist with clinical decision-making.

A number of limitations exist in this systematic review. The first limitation was the authors’ inability to collect all full text and/or English versions of relevant studies. Some studies were inaccessible in English language and some abstracts were not yet published in full text form. As a result, there may have been relevant studies that were not included in this review. Another potential limitation of this review was the author’s decision to exclude studies if they included participants outside of childhood age range (0 to 18 years). This resulted in data on children within the accepted age range being excluded as it could not be dissected from adult populations. Although attempts were made to extract raw data pertaining to participants aged 0 to 18 years, if relevant data could not be isolated in the publication, the study was excluded altogether. Further, inclusion of studies with participants ranging from 0 to 18 years may limit the translation of findings to specific more refined age populations within this range. Publication of de-identified data sets (as is now being requested by many journals) may have been useful for addressing this limitation. The authors decision to record the effects of serial casting across multiple timeframes and introduce these into the meta-analyses was undertaken to enhance the effect size and opportunity for a conclusive result, however this created significant heterogenous samples for some analyses, suggesting a large degree of dissimilarity in the individual study results. To account for the significant heterogeneity, a random effects model was used when appropriate to assess both intra-study sampling errors and between-study variation, resulting in wider confidence intervals and more conservative findings. A different methodological approach to data synthesis and analysis may have led to even stronger findings when examining the effects of serial casting on each of the outcome measures.

Despite the above-mentioned limitations, there are also many strengths to the present review. The methodology conducted was thorough and comprehensive with two to three reviewers involved at every stage, ensuring the reliability of the results from this study. Additionally, this systematic review provided a meta-analytic approach to addressing the study aims whenever possible, which was not done in previously published systematic reviews or overviews investigating the same or similar topics. Another strength of this review was the authors’ decision to ensure quality of the results being synthesised by only including fair to good quality studies for meta-synthesis, and only analyzing outcomes if there was a minimum of five investigations for the meta-synthesis and a minimum of two investigations for meta-analysis. Although a consequence of these methods meant that some data relevant to the effects of serial casting were omitted from analysis and thus from the overall findings of the review, it ensured that the results presented, and conclusions drawn were from accumulated trustworthy sources. The major strength of this review was that it drew conclusions from a detailed meta-synthesis and further quantified findings of previous reviews by including a meta-analysis using data from the stronger methodological quality studies only, when it was possible. No previous systematic review exploring the effects of serial casting on lower limb function in children with CP has previously undertaken a meta-analysis to form conclusions, except for Kelly and colleagues who published a Cochrane protocol in 2008 [25], however this systematic review was withdrawn from publication in 2017 due to being out of date. Since the beginning of 2008, there have been six additional relevant papers added to the empirical literature that were included in our review. More recently Novak and colleagues in 2013 [17], published a broad systematic review supporting the use of serial casting to improve joint range in the lower limbs, however no findings were published in this review regarding the effects of serial casting on functional gait. Therefore, we feel that the present review provides new insights into the functional benefits of serial casting independent of BTX-A.

## Conclusions

Findings from this systematic review suggest that serial casting is effective for increasing passive ankle DF PROM and decreasing hypertonicity in the lower limbs. Additionally, the findings of this review suggest that serial casting coupled with BTX-A in children with CP has a slightly more but significantly favorable effect on ankle DF PROM. Further high-quality research is required to investigate the effects of serial casting with and without pharmacological interventions on other functional lower limb outcomes. Overall, the results of this systematic review support the clinical use of serial casting of the lower limb for improving functional outcomes (i.e. gait via increased ankle DF) in children with CP and suggest that the effects on ankle DF PROM may be slightly stronger when coupled with BTX-A. These findings however, need to be balanced against recent research which showed that small improvements in ankle DF achieved from BTX-A injections to the gastrocnemius (without the addition of serial casting) were offset by potentially harmful effects of deteriorating knee kinematics, moving children with spastic diplegia CP towards crouch gait patterns [34]. Clinicians may use the information gained from the present systematic review when developing individualized treatment plans for children who have CP during shared decision-making consultations clients and their families. Further research investigating the longitudinal effects of serial casting using clearly documented casting protocols and assessment procedures would be valuable to clinicians and researchers.

## Supplementary information

**Additional file 1 Table S1.** Serial Casting and Functional Outcomes of the Lower Limb in Children with CP: Data Extraction Table.

## Data Availability

Detailed summaries of all included studies are provided in Table S1: Supplement File 1_Serial Casting and CP Data Extraction Table.

## Abbreviations

CP: Cerebral Palsy
DF: Dorsiflexion
ROM: Range of Motion
AROM: Active Range of Motion
PROM: Passive Range of Motion
MAS: Modified Ashworth Scale
MTS: Modified Tardieu Scale
GMFM: Gross Motor Function Measure
MD: Mean Difference
SMD: Standardised Mean Difference
SD: Standard Deviation
CI: Confidence Interval
BTX-A: Botulinum Toxin A
SDR: Selective Dorsal Rhizotomy
SEMLS: Single-event multi-level surgeries
ICF: International Classification of Functioning, Disability and Health model
PRISMA: Preferred Reporting Items for Systematic Reviews and Meta-Analyses
CAS: Critical Appraisal Score
IP: Immediately post (serial casting)
ST: Short-term (> 7 days and < 3 months post serial casting)
MT: Mid-term (3 months to > 6 months post serial casting)
LT: Long-term (> 6 months post serial casting)
SC: Serial Casting
P: Pharmacological Intervention
A: Ankle Foot Orthosis / or similar lower limb orthoses
T: Therapy - Physical or Occupational
KPF: Knee Position Flexed
KPE: Knee Position Extended
KPU: Knee Position Unspecified
PRS: Physicians Rating Scale
R1: Resistance 1 (spastic catch)
R2: Resistance 2 (end of range)
OGS: Observational Gait Scale
GMFCS: Gross Motor Function Classification Scale
